# Establishment of a genome‐editing system to create fragrant germplasm in sweet sorghum

**DOI:** 10.1007/s42994-024-00180-6

**Published:** 2024-09-27

**Authors:** Zixiang Cheng, Ke Li, Hongxiu Liu, Xingen Wei, Tao Yin, Xin Xing, Lida Han, Yi Sui

**Affiliations:** 1 State Key Laboratory of Crop Gene Resources and Breeding, Institute of Crop Sciences, Chinese Academy of Agricultural Sciences, Beijing, 100081 China; 2 Biotechnology Research Institute, Chinese Academy of Agricultural Sciences, Beijing, 100081 China

**Keywords:** Sweet sorghum, Genetic transformation, CRISPR/Cas9 editing, Fragrant sweet sorghum

## Abstract

Sorghum, the fifth largest global cereal crop, comprises various types, such as grain, sweet, forage, and biomass sorghum, delineated by their designated end uses. Among these, sweet sorghum (*Sorghum bicolor* (L.) Moench) stands out for its unique versatility, exceptional abiotic stress tolerance and large biomass serving the multi-purpose of high-sugar forage, syrup, and biofuel production. Despite its significance, functional genomic research and biotechnological breeding in sweet sorghum are still in nascent stages, necessitating more efficient genetic transformation and genome-editing techniques. This study unveils Gaoliangzhe (GZ), an elite sweet sorghum variety for heightened resistance to salinity and drought. Through the establishment of an *Agrobacterium tumefaciens*‐mediated genetic transformation and CRISPR/Cas9-based genome-editing system in GZ, a breakthrough is achieved. Using genome-editing technology, we first produced a fragrant sweet sorghum line by targeting the *BETAINE ALDEHYDE DEHYDROGENASE 2* (*SbBADH2*) gene. Our results establish a strong foundation for further functional genomic research and biotechnological breeding of sweet-sorghum varieties.

**Supplementary Information:**

The online version contains supplementary material available at 10.1007/s42994-024-00180-6.

## Dear Editor,

According to a recent report by the Food and Agriculture Organization (FAO) of the United Nations, global soil salinization is intensifying, highlighting an alarming deterioration in global soil resources. Indeed, over 7% of the world’s lands and 20% of irrigated lands are affected by salinization; moreover, the affected arable land is projected to exceed 50% by 2050. Therefore, developing salinity-tolerant crop varieties is crucial to address this issue to offer increased food and silage production in support of the ever-growing global population, thus aligning with the objectives of the United Nations Sustainable Development Goals, specifically, “Zero Hunger” (SDG2) and “Land Life” (SDG15) (Xie and Xu 2019; Singh 2021; Ren et al. 2022; Ge et al. 2023).

Domesticated sorghum is typically classified by end uses: grain, sweet, forage, and biomass sorghum. Sweet sorghum, a variant of sorghum, exhibits high tolerance to various abiotic stress factors, such as saline-alkali, drought, waterlogging, and high temperature conditions. In addition, it shows high water-use efficiency, substantial biomass, and high-sugar content in the stem. As a C4 plant, sweet sorghum produces 2–3 times more biomass per harvest than maize possessing highly efficient photosynthesis and nitrogen-use efficiency (Xie and Xu 2019; Yang et al. 2020; Sun et al. 2023). Consequently, sweet sorghum can thrive in saline-alkali and arid marginal soils without competing with major crops, thereby providing additional food resources and bioenergy (Antonopoulou et al. 2008; Xie and Xu 2019; Zheng et al. 2023a). Despite the identification of key-genes-regulating stem sap and salt tolerance in sweet sorghum, the lack of a robust genetic transformation system and effective genome-editing tools, with limited access to emerging genome-editing technologies, such as CRISPR/Cas9, has hindered rapid progress in sweet-sorghum genetic improvement (Song et al. 2020; Hao et al. 2021; Sun et al. 2023).

Aromatic traits are important for crop breeding. In particular, 2-acetyl-1-pyrroline (2-AP) is the major compound contributing to rice fragrance (Chen et al. 2008). In addition, the *BETAINE ALDEHYDE DEHYDROGENASE 2* (*BADH2*) gene encoding betaine dehydrogenase, which is responsible for 2-AP production and fragrance emission, is a conserved and valuable target for genetic mutations, as observed in fragrant rice, maize, and foxtail millet (Chen et al. 2008; Wang et al. 2021; Zhang et al. 2023b). The integration of genetic transformation and CRISPR/Cas9-based genome-editing technologies represents a promising strategy for plant breeding, revolutionizing crop improvement processes, and enhancing global food security (Gao et al. 2021). Although previous success in generating fragrant lines in grain sorghum (Wheatland), using the CRISPR/Cas9 system, reinforces the potential for creating aromatic varieties of this crop (Zhang et al. 2022), sweet-sorghum fragrant-line production has not been reported.

**Fig. 1:**
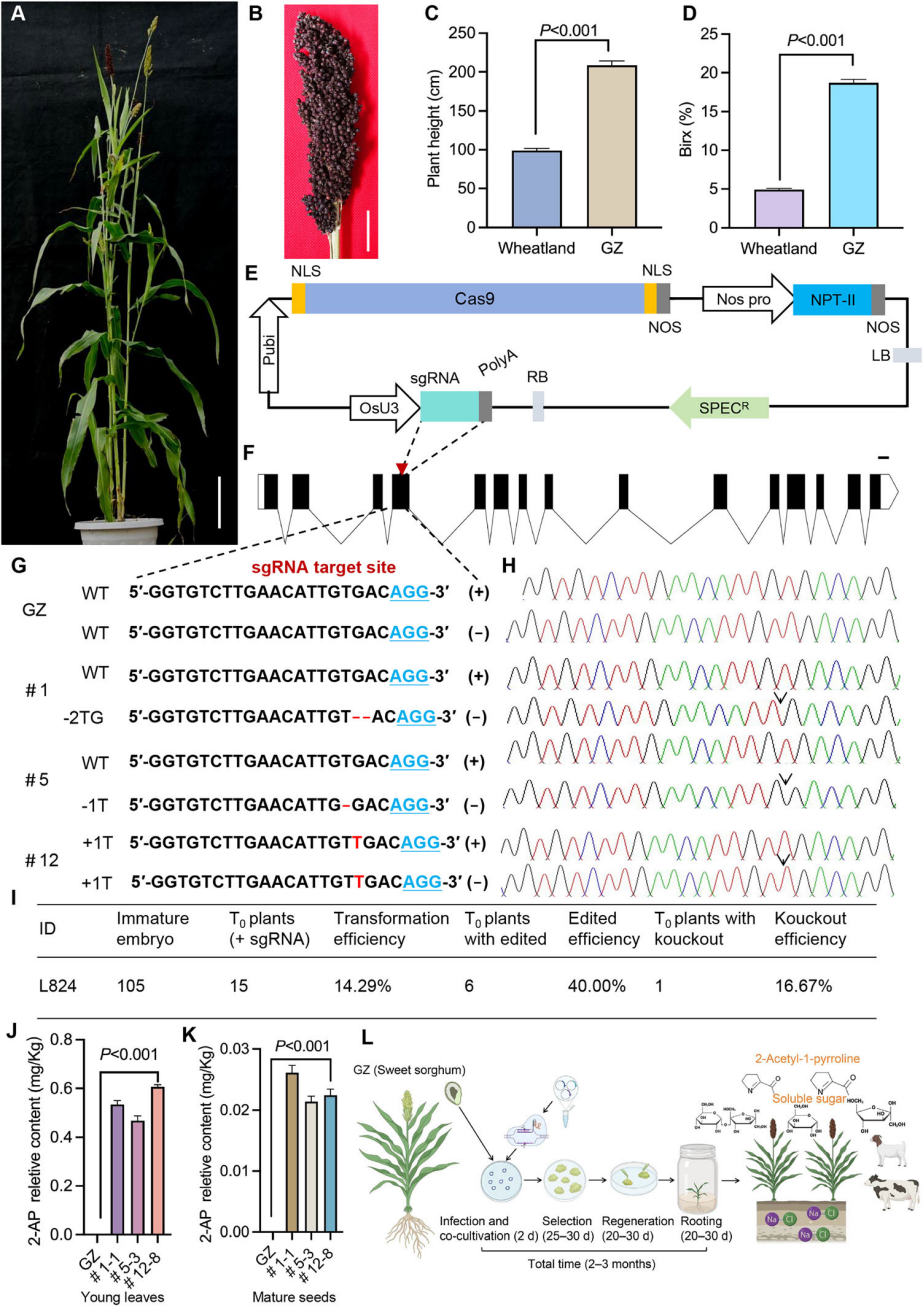
CRISPR/Cas9‐mediated genome-editing system and production of fragrant germplasm in GZ. **A** Pot-grown GZ plant. Scale bar: 15 cm. **B** Morphology of the GZ panicle. Scale bar: 2 cm. **C** Comparison of plant height between the sorghum cultivar Wheatland and elite GZ. **D** Analysis of Brix in the stem of sweet sorghum GZ and grain sorghum Wheatland. *P* values were calculated using the two-tailed Student’s *t* test.** E** Schematic diagram of the CRISPR/Cas9-SbBADH2 vector. **F** *SbBADH2* gene structure. The white box represents the 5′-untranslated region (UTR) and 3′-untranslated region (UTR). The black box and lines represent exons and introns. Red arrowheads mark the target sites. Scale bar: 100 bp. **G** Targeted mutagenesis in the *SbBADH2* gene. The protospacer adjacent motif (PAM) sites are highlighted in blue. Red dashes represent deletions within the targets, and insertions are highlighted by red “T” or “G.” **H** The panel shows Sanger sequencing chromatograms of edited *SbBADH2* alleles. The arrows indicate the edited positions. **I** Transformation and genome-editing efficiency within the T_0_ generation. **J** Relative contents of 2‐AP in the 4‐week‐old leaves. **K** Relative contents of 2‐AP in the dried mature seeds. **L** Schematic workflow of *Agrobacterium*‐mediated genetic transformation and genome-editing system for GZ

Here, we aimed to develop a genetic transformation and genome-editing system for sweet sorghum. The elite variety Gaoliangzhe (referred to as GZ) is recognized for its sweetness akin to sugarcane with a mean stem Brix of 18.76% and large biomass (Fig. 1A–D), and remarkable salinity and drought tolerance (Ren et al. 2022). Therefore, GZ was selected for genome editing. Our focus was on the *SbBADH2* gene, using a CRISPR/Cas9 vector with Cas9 driven by the maize *ubiquitin* promoter, a single guide RNA (sgRNA) targeting the *SbBADH2* gene, driven by the rice *U3* promoter (*OsU3*), and neomycin phosphotransferase II (*NPT-II*), as a selective marker, to establish an effective genome-editing protocol for GZ (Fig. 1E).

We used our *Agrobacterium*-mediated transformation protocol to transfer the CRISPR/Cas9 vector into immature GZ embryos, and obtained 15 transgene-positive T_0_ plants (Table S1). Subsequently, we identified the types of *SbBADH2* gene mutations in these transgene-positive T_0_ plants. Of the 15 T_0_ plants, six showed edited genome at the *SbBADH2* locus, achieving an editing efficiency of 40.00%, and characterized by base deletions and insertions leading to a frameshift or early termination of *SbBADH2* (Fig. 1F–H). Among these six plants, one exhibited a complete knockout mutation, whereas five contained heterozygous mutations, resulting in a knockout efficiency of 16.7% (1/6) (Fig. 1I).

We selected three homozygous lines to analyze 2-AP content. An organoleptic fragrance test revealed a distinct aromatic smell in the leaves collected from 4-week-old edited sweet-sorghum lines when exposed to a 1.7% KOH solution for 15 min. Gas chromatography-mass spectrometry (GC–MS) was used to quantify the concentration of 2-AP in the fresh leaves and mature seeds. Edited plants showed a mean 2-AP content of 0.53 mg/kg and 0.023 mg/kg in leaves and seeds, respectively, which were higher than that recorded for the wild type (Fig. 1J, K). We assessed six agronomic traits of both the edited lines and the wild type including birx, biomass, plant height, heading date, main panicle weight per plant, and thousand‐grain weight, which showed no significant difference between the wild‐type and the edited lines (Fig. S1).

Although, we successfully established a genetic transformation and genome-editing system using CRISPR/Cas9 in the sweet-sorghum variety GZ with a genetic transformation cycle of 2–3 months (Fig. 1L), the scope for enhancement of transformation efficiency and genome-editing precision is enormous. The incorporation of morphogenic genes, such as *Baby Boom* and *Wuschel2* offers the potential to improve transformation efficiency and overcome genotype-specific limitations (Aregawi et al. 2019). Furthermore, optimizing the Cas9 protein, based on the genome-codon usage of GZ and cloning its *U6* or *U3* promoter to drive sgRNA expression, may enhance genome-editing efficiency.

Research by the FAO of the United Nations shows that there are > 1 billion ha of land affected by salt (in 2015; https://www.fao.org/3/i5199e/i5199e.pdf). The use of saline-alkali soils for crop production is an effective way to ensure food security, worldwide (Zhang et al. 2023a). We successfully enhanced 2-AP accumulation in sweet sorghum by mutating the *SbBADH2* gene. Aromatic quality enhances the appeal to livestock such as cattle, sheep, and rabbits, elevating the forage quality of sweet sorghum. In addition, aromatic sweet sorghum grains may potentially boost the competitiveness in the brewing industry. Products such as wine and vinegar derived from fragrant sweet sorghum may offer superior taste profiles to satisfy the preferences of a wider consumer base (Fig. 1K and Fig S1E, F). Nevertheless, further evaluation is required to ascertain whether there are any alterations in the susceptibility of fragrant sweet sorghum to pests or diseases.

In conclusion, we successfully established a CRISPR/Cas9-based genome-editing technique for the sweet-sorghum variety GZ and developed fragrant sweet sorghum for the first time. This finding paves the way for further research into stress-resistance genes and facilitating transgenic breeding of this important crop.

## Materials and methods

### Plant material and growth conditions

Sweet sorghum was grown in an experimental field in Haidian, Beijing, China (39°54′ 20″ N, 116°25′ 29″ E), as well as in pots (40 cm × 50 cm) within a greenhouse. Greenhouse-cultivated plants were grown under a 14 h photoperiod, with day/night temperatures set to 32/22 °C and average relative humidity of 40–50%.

### Vector construction and genetic transformation of sweet sorghum

Construction of CRISPR/Cas9 vector and genetic transformation protocol for GZ were carried out via the previous method and slightly modified (Miao et al. 2013; Zheng et al. 2023b). See the supplementary material for the detailed protocol.

### Identifying the mutation in the *SbBADH2* gene

Total DNA was extracted from transgenic plants utilizing the CTAB protocol. PCR was performed using 2 × Phanta Flash Master Mix (Dye Plus) (P520-01, Vazyme Biotech Co., Ltd., Nanjing, China). Primer sequences are listed in Table S2.

### Measurement of 2-AP content in sweet sorghum

Quantification of 2-AP was performed as the previously described protocol (Zhang et al. 2022). The amount of 2-AP was measured by gas chromatography-mass spectrometry (GC-MS) (7890-7000B; Agilent, Beijing, China). The capillary column used was HP-5MS UI (30 m × 0.25 mm × 0.25 μm).

## Supplementary Information

Below is the link to the electronic supplementary material.

Supplementary file1 (XLSX 12 KB)

Supplementary file2 (DOCX 246 KB)

## Data Availability

The data are available on request from the corresponding author.

## References

[CR1] Antonopoulou G, Gavala H, Skiadas V, Angelopoulos K, Lyberatos G (2008) Biofuels generation from sweet sorghum: fermentative hydrogen production and anaerobic digestion of the remaining biomass. Bioresour Technol 99:110–119. 10.1016/j.biortech.2006.11.048 17257834 10.1016/j.biortech.2006.11.048

[CR2] Chen S, Yang Y, Shi W, Ji Q, He F, Zhang Z, Cheng Z, Liu X, Xu M (2008) *Badh2*, encoding betaine aldehyde dehydrogenase, inhibits the biosynthesis of 2-acetyl-1-pyrroline, a major component in rice fragrance. Plant Cell 20:1850–1861. 10.1105/tpc.108.058917 18599581 10.1105/tpc.108.058917 PMC2518245

[CR3] Gao C (2021) Genome engineering for crop improvement and future agriculture. Cell 184:1621–1635. 10.1016/j.cell.2021.01.005 33581057 10.1016/j.cell.2021.01.005

[CR4] Ge F, Xie P, Wu Y, Xie Q (2023) Genetic architecture and molecular regulation of sorghum domestication. aBIOTECH 4:57–71. 10.1007/s42994-022-00089-y 37220542 10.1007/s42994-022-00089-y PMC10199992

[CR5] Hao H, Li Z, Leng C, Lu C, Luo H, Liu Y, Wu X, Liu Z, Shang L, Jing H (2021) Sorghum breeding in the genomic era: opportunities and challenges. Theor Appl Genet 134:1899–1924. 10.1007/s00122-021-03789-z 33655424 10.1007/s00122-021-03789-z PMC7924314

[CR6] Miao J, Guo D, Zhang J, Huang Q, Qin G, Zhang X, Wan J, Gu H, Qu L (2013) Targeted mutagenesis in rice using CRISPR-Cas system. Cell Res 23:1233–1236. 10.1038/cr.2013.123 23999856 10.1038/cr.2013.123 PMC3790239

[CR7] Ren G, Yang P, Cui J, Gao Y, Yin C, Bai Y, Zhao D, Chang J (2022) Multiomics analyses of two sorghum cultivars reveal the molecular mechanism of salt tolerance. Front Plant Sci 13:886805. 10.3389/fpls.2022.886805 35677242 10.3389/fpls.2022.886805 PMC9168679

[CR8] Singh A (2021) Soil salinization management for sustainable development: a review. J Environ Manag 277:111383. 10.1016/j.jenvman.2020.111383 10.1016/j.jenvman.2020.111383 33035935

[CR9] Song Y, Li J, Sui Y, Han G, Zhang Y, Guo S, Sui N (2020) The sweet sorghum *SbWRKY50* is negatively involved in salt response by regulating ion homeostasis. Plant Mol Biol 102:603–614. 10.1007/s11103-020-00966-4 32052233 10.1007/s11103-020-00966-4

[CR10] Sun X, Zheng HX, Li S, Gao Y, Dang Y, Chen Z, Wu F, Wang X, Xie Q, Sui N (2023) MicroRNAs balance growth and salt stress responses in sweet sorghum. Plant J 113:677–697. 10.1111/tpj.16065 36534087 10.1111/tpj.16065

[CR11] Wang Y, Liu X, Zheng X, Wang W, Yin X, Liu H, Ma C, Niu X, Zhu J, Wang F (2021) Creation of aromatic maize by CRISPR/Cas. J Integr Plant Biol 63:1664–1670. 10.1111/jipb.13105 33934500 10.1111/jipb.13105

[CR13] Xie Q, Xu Z (2019) Sustainable agriculture: from sweet sorghum planting and ensiling to ruminant feeding. Mol Plant 12:603–606. 10.1016/j.molp.2019.04.001 31002980 10.1016/j.molp.2019.04.001

[CR14] Yang Z, Li J, Liu L, Xie Q, Sui N (2020) Photosynthetic regulation under salt stress and salt-tolerance mechanism of sweet sorghum. Front Plant Sci 10:1722. 10.3389/fpls.2019.01722 32010174 10.3389/fpls.2019.01722 PMC6974683

[CR16] Zhang D, Tang S, Xie P, Yang D, Wu Y, Cheng S, Du K, Xin P, Chu J, Yu F, Xie Q (2022) Creation of fragrant sorghum by CRISPR/Cas9. J Integr Plant Biol 64:961–964. 10.1111/jipb.13232 35142064 10.1111/jipb.13232

[CR17] Zhang H, Yu F, Xie P, Sun S, Qiao X, Tang S, Chen C, Yang S, Mei C, Yang D, Wu Y, Xia R, Li X, Lu J, Liu Y, Xie X, Ma D, Xu X, Liang Z, Feng Z, Huang X, Yu H, Liu G, Wang Y, Li J, Zhang Q, Chen C, Ouyang Y, Xie Q (2023a) A Gγ protein regulates alkaline sensitivity in crops. Science 379:8416. 10.1126/science.ade8416 10.1126/science.ade8416 36952416

[CR18] Zhang Y, He Q, Zhang S, Man X, Sui Y, Jia G, Tang S, Zhi H, Wu C, Diao X (2023b) De novo creation of popcorn-like fragrant foxtail millet. J Integr Plant Biol 65:2412–2415. 10.1111/jipb.13556 37565564 10.1111/jipb.13556

[CR19] Zheng H, Dang Y, Sui N (2023a) Sorghum: a multipurpose crop. J Agric Food Chem 71:17570–17583. 10.1021/acs.jafc.3c04942 37933850 10.1021/acs.jafc.3c04942

[CR20] Zheng H, Gao Y, Sui Y, Dang Y, Wu F, Wang X, Zhang F, Du X, Sui N (2023b) R2R3 MYB transcription factor *SbMYBHv33* negatively regulates sorghum biomass accumulation and salt tolerance. Theor Appl Genet 136:5. 10.1007/s00122-023-04292-3 36656365 10.1007/s00122-023-04292-3

