# Supplementary material for: Establishment of a genome‐editing system to create fragrant germplasm in sweet sorghum
Source: aBIOTECH. 2024 Sep 27;5(4):502–6. doi: 10.1007/s42994-024-00180-6 (PMC11624148; doi:10.1007/s42994-024-00180-6)
Supplement: Supplementary file 2 — Supplementary file2 (DOCX 246 KB) [file 42994_2024_180_MOESM2_ESM.docx]

**Supplementary information**

**
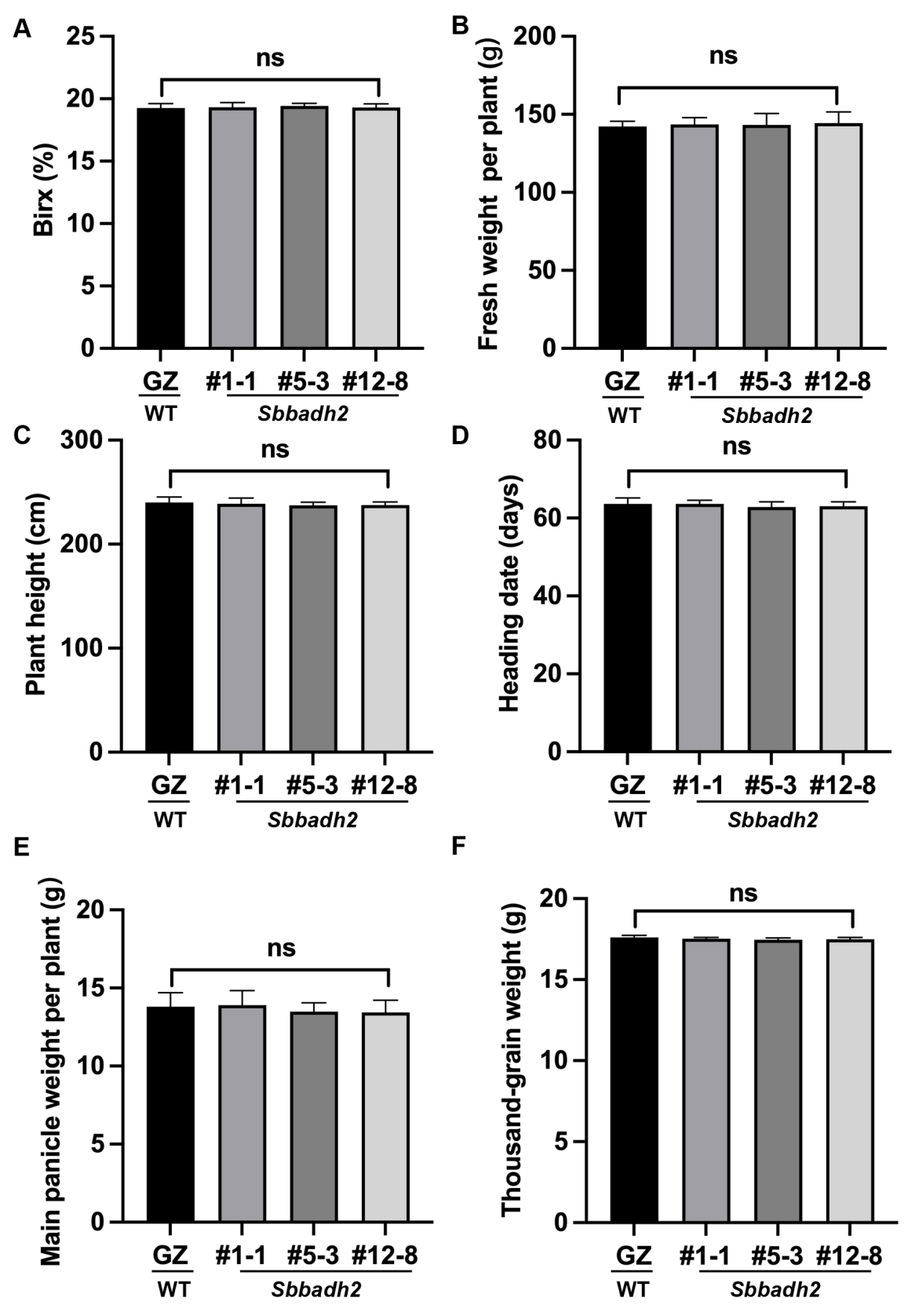
**

**Fig.S1. Comparison of agronomic traits between *sbbadh2* and wild type.** The traits evaluated include Birx (**A**), biomass (20 days after flowering) (**B**), plant height (**C**), heading date (**D**), main panicle weight per plant (**E**), and thousand‐grain weight (**F**). The data presented are means ± standard deviations based on 20 replicates (n = 20). *P*-values were calculated using the two-tailed student's *t*-test.

**Extended Materials and Methods**

**Construction of CRISPR/Cas9 gene editing vector**

A CRISPR/Cas9 vector was designed with a 20 bp target site specific to the *SbBADH2* gene. Oligonucleotide DNA primers (*SbBADH2-target-F/SbBADH2-target-R*), consisting a 20 nt sgRNA sequence and a 30 bp adaptor, were synthesized for the *SbBADH2* gene within the PCas9-P vector. For primer annealing, 1μl of *SbBADH2-target-F* and 1μl *SbBADH2-target-R* were added to 8 μl of ddH_2_O, and the mixture underwent gradual cooling at a rate of 0.1℃/s to 15℃. The PCas9-P vector was linearized using the *Aar*I restriction enzyme. The annealed product was then subjected to an infusion reaction using In-Fusion HD Cloning Plus kit (Catalog#: 638910; Takara Bio, USA) and subsequently introduced into *E.coli* cells to obtain suitable clones for sequencing. The correct vector was introduced into the *Agrobacterium* strain EHA105 (*Agrobacterium tumefaciens*) and subsequently confirmed by re-transforming it into *E.coli*. Following this confirmation, *Agrobacterium*-mediated genetic transformation was performed.

**Sweet sorghum transformation**

Immature seeds (12–14 days post-pollination) were harvested, their husks removed and then sterilized in 70% (v/v) ethanol for 5 minutes, followed by treatment with 12% (v/v) bleach for 10-15 minutes. The seeds were rinsed three times with autoclave water. Immature embryos measuring 1.0-1.5 mm in length were isolated from the seeds and placed into an infection liquid medium composed of 0.44 g/L Murashige–Skoog salts, 1×B_5_ vitamins, 68 g/L sucrose, 36 g/L glucose, 1 g/L asparagine, 1 g/L casamino acids, 0.2 g/L cysteine, 2 mg/L 2,4-dichlorophenoxyacetic acid, 200 μM acetosyringone, pH 5.2. Positive EHA105 cells were cultured overnight in YEB medium (5 g/L beef extract, 5 g/L peptone, 1 g/L yeast extract, 5 g/L sucrose, 10 mM magnesium sulfate, pH 7.0) until the optical density at 600 nm (OD 600) reached 1.0, preparing them for use. For infection, approximately 100-200 immature embryos underwent heat treatment (43℃ for 3 minutes) before being inoculated with 1 ml of the bacterial cell suspension for about 5 minutes. After infection, the embryos were transferred to a co-cultivation medium (infection medium with 8 g/L agarose), positioned with the scutellum facing up, and co-cultivated at 22℃in the dark for 3 days. Following co-cultivation, the embryos were subcultured on CIM (4.33 g/L Murashige–Skoog salts, 1×B_5_ vitamins, 30 g/L sucrose, 0.2 g/L asparagine, 1 g/L casamino acids, 0.1 g/L myo-inositol, 8 g/L agarose, 2 mg/L 2,4-dichlorophenoxyacetic acid, pH 5.7). Resting medium containing 250 mg/L Timentin at 28℃ in the dark for 6-7 days. They were then transferred to CIM selection medium containing 50 mg/L paromomycin for approximately 10 days. An additional 20 days of selection in the same medium was conducted until resistant calli were formed, which were then transferred to SM medium (CIM medium without 2,4-dichlorophenoxyacetic acid, containing 2 mg/L BAP, 250 mg/L timentin, 50 mg/L paromomycin, 8 g/L agarose, pH 5.7) at 28℃ under 16 hours light and 8 hours dark conditions for 2-3 weeks. Elongated shoots measuring 1-3 cm in length were then transferred to rooting medium (half-strength Murashige–Skoog basal salt with vitamins, 30 g/L sucrose, 0.1 g/L myo-inositol, 2.6 g/L Gelzan (gellan gum), pH 5.6) for root development. Putative transgenic plants with healthy roots were then transferred to pots prior to being moved to the field.
